# Characteristics and management of HIV-1-infected pregnant women enrolled in a randomised trial: differences between Europe and the USA

**DOI:** 10.1186/1471-2334-7-60

**Published:** 2007-06-20

**Authors:** Marie-Louise Newell, Sharon Huang, Simona Fiore, Claire Thorne, Laurent Mandelbrot, John L Sullivan, Robert Maupin, Isaac Delke, D Heather Watts, Richard D Gelber, Coleen K Cunningham, the PACTG 316 Study Team

**Affiliations:** 1 Centre of Paediatric Epidemiology and Biostatistics, Institute of Child Health, University College London, UK; 2 Centre for Biostatistics in AIDS Research, Harvard School of Public Health, Boston, USA; 3 Service de Gynecologie-Obstetrique, APHP Hopital Louis Mourier, F-75701 Colombes, Universite Diderot, Paris 7, and Inserm, U822, IFR69, F-94276, France; 4 Department of Pediatrics and Molecular Medicine, University of Massachusetts Medical School, Worcester, USA; 5 Department of Obstetrics and Gynecology, Louisiana State University Health Sciences Center, New Orleans, USA; 6 Department of Obstetrics and Gynecology, University of Florida College of Medicine, Jacksonville, USA; 7 Pediatric, Adolescent, and Maternal AIDS Branch, National Institute of Child Health and Human Development, Bethesda, USA; 8 Department of Pediatrics, Duke University Medical Center, Durham, USA

## Abstract

**Background:**

Rates of mother-to-child transmission of HIV-1 (MTCT) have historically been lower in European than in American cohort studies, possibly due to differences in population characteristics. The Pediatric AIDS Clinical Trials Group Protocol (PACTG) 316 trial evaluated the effectiveness of the addition of intrapartum/neonatal nevirapine in reducing MTCT in women already receiving antiretroviral prophylaxis. Participation of large numbers of pregnant HIV-infected women from the US and Western Europe enrolling in the same clinical trial provided the opportunity to identify and explore differences in their characteristics and in the use of non-study interventions to reduce MTCT.

**Methods:**

In this secondary analysis, 1350 women were categorized according to enrollment in centres in the USA (n = 978) or in Europe (n = 372). Factors associated with receipt of highly active antiretroviral therapy and with elective caesarean delivery were identified with logistic regression.

**Results:**

In Europe, women enrolled were more likely to be white and those of black race were mainly born in Sub-Saharan Africa. Women in the US were younger and more likely to have previous pregnancies and miscarriages and a history of sexually transmitted infections.

More than 90% of women did not report symptoms of their HIV infection; however, more women from the US had symptoms (8%), compared to women from Europe (4%). Women in the US were less likely to have HIV RNA levels <400 copies/ml at delivery than women enrolling in Europe, and more likely to receive highly active antiretroviral therapy, and to start therapy earlier in pregnancy. The elective caesarean delivery rate in Europe was 61%, significantly higher than that in the US (22%). Overall, 1.48% of infants were infected and there was no significant difference in the rate of transmission between Europe and the US despite the different approaches to treatment and delivery.

**Conclusion:**

These findings confirm that there are important historical differences between the HIV-infected pregnant populations in Western Europe and the USA, both in terms of the characteristics of the women and their obstetric and therapeutic management. Although highly active antiretroviral therapy predominates in pregnancy in both settings now, population differences are likely to remain.

**Trial registration:**

NCT00000869

## Background

Rates of mother-to-child transmission of HIV-1 (MTCT) have historically been lower in European than in American cohort studies [1-8]. It has been suggested that this was likely to be related to differences in population characteristics [9,10]. Although in the 1980s and early 1990s the HIV epidemic among pregnant women in Europe was mainly associated with injecting drug use (IDU) [11,12], since the mid-1990s there has been a shift towards heterosexually-acquired HIV, largely in women of sub-Saharan origin [12,13]. Generally, health care services and in particular antenatal care is readily available to these populations in Western Europe. In the USA, there has been a similar trend, with heterosexual acquisition overtaking IDU as the main transmission category among women with AIDS in 1995 [14]; in 2004, 78% of HIV infections among women were due to heterosexual contact, 20% to IDU and 2% to other routes [14]. Differences in health care provision and standards of care may have led to differences between these two geographical regions in the availability and uptake of various interventions available for HIV infected pregnant women [15-19].

The PACTG 316 trial was a placebo-controlled randomised trial to evaluate the additional value of single dose nevirapine (sdNVP) at delivery in reducing MTCT in women already receiving antiretroviral prophylaxis [20]. The trial was initiated in the USA in 1997 and extended through collaboration with two large ongoing European perinatal cohort studies, the French Perinatal Cohort Study (ANRS 083) and the European Collaborative Study [21]. The trial was stopped prematurely in June 2000 after the results of a planned interim analysis indicated a lower than anticipated overall MTCT rate (1.5%), without a significant difference between the sdNVP and the placebo arms [20].

We here take the opportunity to identify and explore differences in the characteristics of HIV infected pregnant women enrolling in the trial, and in the use of interventions to reduce MTCT between Europe and USA, in particular, the use of highly active antiretroviral therapy (HAART) and elective caesarean delivery.

## Methods

The PACTG 316 trial was initiated in the United States (including Puerto Rico) in May, 1997. Between May 1997 and June 2000, centres collaborating in France, Italy, Spain, Sweden, UK, Belgium, Germany, Switzerland, Holland, Denmark, Bahamas and Brazil were invited to join the PACTG 316 trial. The methods of this randomized, blinded trial have been fully described elsewhere [20]. In brief, HIV-infected pregnant women were initially enrolled in the trial after 28 weeks of gestation (later reduced to 20 weeks), with written informed consent. They were randomised to receive either oral nevirapine (200 mg at onset of labour) or placebo; their infants received the same study drug (infant 2 mg/kg oral nevirapine or placebo) 48–72 hours after birth. All clinicians were encouraged to offer all HIV-infected pregnant women at least a regimen of prophylactic zidovudine monotherapy in line with the ACTG 076 protocol [22]. Women were allowed to receive any combination of licensed antiretroviral drugs in pregnancy as prescribed by their clinician, with the exception of non-nucleoside reverse transcriptase inhibitors.

In all participating centres, HIV-infected women were systematically identified during pregnancy, according to local practice. At enrolment, baseline information including socio-demographic characteristics, obstetric history, history of and current use of antiretroviral therapy (ART), results from screening for sexually transmitted infections (STI) (gonorrhea, chlamydia, human papilloma virus, herpes simplex, syphilis and trichomonas vaginalis) and clinical history were collected using standardized forms. Clinical and laboratory evaluations, including CD4 lymphocyte count and HIV RNA quantification were carried out at enrolment, delivery and post-partum. CD4 counts and viral load measurements were carried out locally in laboratories which were certified by ACTG or other regional quality assurance programme. Infants had clinical and laboratory information collected at birth, and subsequently according to the protocol.

### Definitions

In this analysis, we compared all mother-child pairs enrolled in the European centres participating in the trial with those enrolled in the sites in the USA and Puerto Rico. Mode of acquisition of HIV infection was assessed on the basis of self-report; no illicit drug screening was carried out. Gestational age was estimated with ultrasound performed at less than 20 weeks gestation or use of date of last menstrual period that corresponded with uterine size. Caesarean deliveries taking place before the onset of labour and before rupture of membranes were classified as elective caesarean deliveries, with all other caesarean deliveries classified as emergency procedures regardless of indication. Low birth weight was defined as birth weight <2500 g. Classification of type of antenatal ART was on the basis of the most potent therapy received. ART was classified as monotherapy if a single nucleoside was administered at a time, as dual therapy if any two nucleosides were administered and as HAART if a protease inhibitor plus two other drugs (excluding NNRTIs as this was an exclusion criteria for the trial) were administered. Undetectable viral load was defined as having HIV RNA levels below 400 copies/ml. Symptomatic HIV disease was defined as being in the Centers for Disease Control (CDC) disease category B and C.

### Statistical analysis

For univariate comparisons among women between Europe and USA, two-sided tests were performed using χ^2 ^test or Fisher exact tests for discrete outcomes, and Wilcoxon test for continuous outcomes. For the multivariate comparisons, logistic regression analysis was used to obtain odds ratios (OR), adjusted odds ratios (AOR) and 95% confidence intervals (95% CI). Statistical software SAS (SAS Institute, Cary, North Carolina, USA) was used for the statistical analysis.

## Results

A total of 1350 women enrolled in the trial had delivered by the time the trial was stopped, 978 (72%) from the USA and 372 (28%) from Europe. Table 1 presents socio-demographic and most likely mode of acquisition of HIV infection by continent. There was a significantly higher proportion of white women in the European centres than in the USA. Nearly half of the women from European centres were black; most had been born in sub-Saharan Africa. As Table 1 shows, there were significant differences between the two populations with regard to history of reproductive health, with women in the USA having a higher prevalence of previous miscarriages (28% versus 13%) and higher parity compared to those in Europe, who had a higher prevalence of history of pregnancy termination (48% versus 27% in the USA). Substantially and significantly fewer women enrolling in Europe had a history of STI compared with those in the USA.

**Table 1:** Characteristics of HIV-infected pregnant women in PACTG 316, USA versus Europe

**Characteristics**	**USA**	**Europe**	**Total**	p-value*
**Total **mothers delivered	978	372	1350	
**Median age: **entry (IQR)	27.2 (22.9–31.8)	30 (26.9–34)	28.1 (23.9–32.4)	<0.0001
**Ethnicity**				<0.0001
White	119 (12%)	190 (51%)	309 (23%)	
Black	621 (64%)	172 (46%)	793 (59%)	
Hispanic	223 (23%)	8 (2%)	231 (17%)	
Other	15 (1.5%)	2 (0.5%)	17 (1%)	
**Parity**				0.0001
0–1	161 (17%)	80 (22%)	241 (18%)	
2–3	399 (41%)	168 (45%)	567 (42%)	
4–5	267 (27%)	97 (26%)	364 (27%)	
≥6	151 (15%)	27 (7%)	178 (13%)	
**Previous miscarriages**				<0.0001
0	707 (72%)	325 (87%)	1032 (76%)	
1–2	247 (25%)	44 (12%)	291 (22%)	
3–6	24 (2.5%)	3 (1%)	27 (2%)	
**Previous terminations**				<0.0001
0	710 (73%)	192 (52%)	902 (67%)	
1–2	220 (22%)	149 (40%)	369 (27%)	
3–4	41 (4%)	28 (7%)	69 (5%)	
5–12	7 (1%)	3 (1%)	10 (1%)	
**Timing of HIV diagnosis**				<0.0001
Pre-pregnancy	539 (55%)	259 (70%)	798 (59%)	
Antenatal	439 (45%)	112 (30%)	551 (41%)	
**Mode of acquisition^#^**				
Sexual with HIV at risk	336 (34%)	44 (12%)	380 (28%)	<0.0001
Sexual with HIV positive	214 (22%)	105 (28%)	319 (24%)	0.0623
Sexual with HIV unknown	716 (73%)	162 (44%)	878 (65%)	<0.0001
Current IDU	12 (1%)	11 (3%)	23 (2%)	0.0331
Previous IDU	68 (7%)	35 (9%)	103 (8%)	0.1340
Blood transfusion	20 (2%)	7 (2%)	27 (2%)	0.9999
Occupational risk	22 (2%)	3 (1%)	25 (2%)	0.1108
Other risk factor	44 (4%)	8 (2%)	52 (4%)	<0.0001
Unknown risk factor	47 (5%)	46 (12%)	93 (7%)	0.0401
**STD history**				<0.0001
Yes	280 (29%)	19 (5%)	299 (22%)	
No	450 (46%)	353 (95%)	803 (60%)	
Unknown	248 (25%)	0 (0)	248 (18%)	

^# ^Some women reported multiple risk factors

* for the difference USA versus Europe

In terms of HIV-related characteristics, although there were no significant differences between continents with regard to immunological status at study entry and at delivery, women from the USA were more likely to have symptomatic HIV disease (Table 2). Very similar proportions in the two settings had HIV RNA levels below 400 copies/ml at study entry; however, by the time of delivery there was a significant difference between geographic areas, with fewer women in the US having HIV RNA below 400 copies/ml and an overall higher median in the US versus Europe. In a sub-analysis of the 259 women from European and the 539 women from USA sites who had been diagnosed with HIV infection prior to their current pregnancy, almost identical proportions (182/259, 70% and 357/539, 66%) were already receiving ART at enrolment. Among the 606 women in the USA who started ART in pregnancy, the median gestational age at initiation was 18 weeks (range, 14–25 weeks), substantially earlier than the median of 27 weeks (range 18–32) among the 190 women in Europe starting therapy antenatally.

**Table 2:** Characteristics of HIV infected pregnant women, USA versus Europe: disease progression and treatment

**Characteristic**	**USA**	**Europe**	**Overall**	**P-value**
**Median CD4 count: entry (IQR)**	437 (293–600)	420 (299–568)	430 (294–592)	0.6958
<200 cells per ml	124 (13%)	37 (10%)	161 (12%)	
200–499	294 (30%)	132 (35%)	426 (32%)	
≥500	560 (57%)	203 (55%)	763 (57%)	
**Median HIV RNA: entry (IQR)**	581 (274–3635)	700 (200–4740)	594 (243–3845)	0.1225
≤400 copies/ml	424 (43%)	142 (38%)	566 (42%)	
400–2,499	252 (26%)	92 (24%)	344 (25%)	
2,500–10,000	143 (15%)	62 (17%)	205 (15%)	
>10,000	138 (14%)	57 (15%)	195 (14%)	
**Median CD4 count: delivery (IQR)**	480 (308–658)	444 (320–632)	469 (310–653)	0.6849
<200 cells/ml	103 (12%)	26 (8%)	129 (11%)	
200–499	233 (27%)	109 (34%)	342 (29%)	
≥500	531 (61%)	187 (58%)	718 (60%)	
**Median HIV RNA: delivery (IQR)**	405 (237–2638)	200 (68–1300)	400 (200–2300)	0.0001
≤400 copies/ml	450 (50%)	176 (64%)	626 (53%)	
400–2,499	220 (24%)	49 (18%)	269 (23%)	
2,500–10,000	115 (13%)	36 (14%)	151 (13%)	
>10,000	116 (13%)	16 (6%)	132 (11%)	
**Maternal ARV use**				<0.0001
HAART	448 (46%)	119 (32%)	567 (42%)	
Dual therapy	342 (35%)	132 (35%)	474 (35%)	
Monotherapy	182 (19%)	119 (32%)	301 (22%)	
No ARV	6 (0.6%)	2 (0.5%)	8 (0.6%)	
**Timing of ARV initiation**				0.0001
Pre-pregnancy	357 (37%)	182 (49%)	539 (40%)	
During pregnancy	606 (63%)	190 (51%)	796 (60%)	
**Symptomatic HIV disease at entry**				0.0194
Yes	74 (8%)	15 (4%)	89 (7%)	
No	904 (92%)	357 (96%)	1261 (93%)	

Median gestational age at delivery was 38.4 weeks (37.3–39.6) and 38.1 weeks (37.1–39.0), and median birth weight was 3077 g and 3020 g in the USA and Europe, respectively. The prevalence of low birth weight was similar in both continents, at 12% in the US (n = 122) and 14% in Europe (n = 54). Mode of delivery varied significantly by geographic area (p < 0.0001). In the USA, the elective caesarean delivery rate was 22% (n = 212), the emergency caesarean delivery rate was 17% (n = 169) and the vaginal delivery rate was 61% (n = 597); respective rates in Europe were 61% (n = 228), 18% (n = 65) and 21% (n = 79).

Subsequent analyses focused on geographic differences in the use of HAART in pregnancy and of elective caesarean delivery. Table 3 presents the results of univariable and multivariable logistic regression analyses to identify factors associated with likelihood of receiving HAART in pregnancy. Univariably, geographic area, low CD4 count and undetectable viral load at entry and at delivery were associated with receipt of HAART. In analyses adjusting for ethnicity, mode of delivery and immunological and virological status, women from the USA were nearly twice as likely to receive HAART as women from Europe, whilst those with CD4 counts ≥ 400 cells/ml and detectable viral load at trial entry were significantly less likely to receive HAART in pregnancy. Stratified analyses for the two geographic areas identified the same risk factors, with odds ratios of similar magnitudes.

**Table 3:** Factors associated with antenatal HAART use

	**N (%)**	**OR (95% CI)**	**AOR (95% CI)**	**p-value**
**Ethnicity**				
White	309 (23)	1.0	1.0	
Black	793 (59)	1.06 (0.82, 1.39)	0.84 (0.62, 1.13)	0.2470
Other	248 (18)	1.15 (0.82, 1.62)	0.96 (0.66, 1.39)	0.8374
**Geographic area**				
Europe	372 (28)	1.0	1.0	
USA	978 (72)	1.79 (1.39, 2.30)	1.82 (1.40, 2.38)	<0.0001
**Mode of delivery**				
Vaginal delivery or non-elective caesarean	910 (67)	1.0	1.0	
Elective caesarean	440 (33)	0.80 (0.64, 1.01)	0.99 (0.76, 1.28)	0.9366
**CD4 count (entry)**				
< 400 cells per ml	586 (43)	1.0	1.0	
≥400 cells per ml	764 (57)	0.43 (0.35, 0.54)	0.36 (0.28, 0.46)	<0.0001
**CD4 count (delivery)**				
< 400 cells per ml	543 (40)	1.0	1.0	
≥400 cells per ml	807 (60)	0.49 (0.40, 0.62)	0.76 (0.56, 1.03)	0.0757
**Viral load (entry)**				
Undetectable	570 (44)	1.00	1.0	
Detectable	740 (56)	0.58 (0.46, 0.72)	0.48 (0.38, 0.61)	<0.0001
**Viral load (delivery)**				
Undetectable	626 (53)	1.0	1.0	
Detectable	555 (47)	0.79 (0.62, 0.99)	0.97 (0.71, 1.31)	0.8197

In addition to use of antiretroviral drugs in pregnancy and avoidance of breastfeeding, elective caesarean delivery is the other key PMTCT intervention. Further logistic regressions were therefore carried out for likelihood of having an elective caesarean versus other delivery modes (emergency caesarean or vaginal delivery) (Table 4). Delivery in centres in the USA was associated with an adjusted 82% reduced odds of elective caesarean delivery compared with delivery in Europe. Black women were 33% less likely to deliver by elective caesarean than white women, and those receiving two or more antiretroviral drugs antenatally were less likely to have an elective caesarean delivery compared with women receiving monotherapy or no ART (Table 4). As there were distinct differences between the continents regarding many of these explanatory variables (Tables 1 and 2), stratified analyses were carried out for each geographic area separately. Among the women from the USA, none of the explanatory variables in Table 3 were associated with elective caesarean delivery univariably [data not shown]. In Europe, black versus white ethnicity was associated with a reduced likelihood of elective caesarean, with borderline significance (AOR 0.65, 95% CI 0.42–0.99, p = 0.048), and dual therapy and HAART with around a two-thirds reduced likelihood of elective caesarean versus no therapy or monotherapy (AORs 0.35 [0.21–0.60] p < 0.0001; 0.41 [0.24–0.72] p = 0.0015 respectively).

**Table 4:** Factors associated with likelihood of delivery by elective caesarean

	**N (%)**	**OR (95% CI)**	**AOR (95% CI)**	**p-value**
**Ethnicity**				
White	309 (23)	1.0	1.0	
Black	793 (59)	0.41 (0.31, 0.53)	0.67 (0.49, 0.91)	0.0107
Other	248 (18)	0.40 (0.28, 0.57)	0.89 (0.60, 1.32)	0.5490
**Geographic area**				
Europe	372 (28)	1.0	1.0	
USA	978 (72)	0.18 (0.14, 0.23)	0.18 (0.14, 0.24)	0.0001
**Antenatal ART**				
None/monotherapy	309 (23)	1.0	1.0	
Dual therapy	474 (35)	0.51 (0.38, 0.69)	0.57 (0.41, 0.78)	0.0006
HAART	567 (42)	0.54 (0.41, 0.72)	0.70 (0.52, 0.96)	0.0263
**CD4 count (entry)**				
< 400 cells per ml	586 (43)	1.0	1.0	
≥400 cells per ml	764 (57)	1.06 (0.84, 1.33)	1.08 (0.84, 1.39)	0.5371
**CD4 count (delivery)**				
< 400 cells per ml	543 (40)	1.0	1.0	
≥400 cells per ml	807 (60)	1.04 (0.83, 1.31)	1.10 (0.86, 1.42)	0.4486
**Viral load (entry)**				
Undetectable	570 (44)	1.00	1.0	
Detectable	740 (56)	0.97 (0.77, 1.23)	0.83 (0.65, 1.07)	0.1571
**Viral load (delivery)**				
Undetectable	626 (53)	1.0	1.0	
Detectable	555 (47)	0.82 (0.64, 1.05)	0.90 (0.69, 1.17)	0.4308

The primary outcome measure of the PACTG 316 trial was detection of HIV infection in the infants. Overall there was a very low rate of MTCT at 1.48% [95% CI 0.91–2.28] (20 vertical transmissions; USA 1.64% [95% CI 0.94–2.64] and Europe 1.08% [0.29–2.73]).

## Discussion

There were distinct differences between the HIV-infected pregnant women enrolling in the PACTG 316 trial in the USA (accounting for nearly three-quarters of the total mother-child pairs in the trial) compared with the European centres. Black women of African-American ethnicity predominated in the US setting, with a further quarter of the women enrolling of Hispanic ethnicity, and only 12% being non-Hispanic white. Those recruited in Europe were almost equally divided between white European and non-white women, the vast majority of whom were of African origin who had arrived in Europe as asylum-seekers, refugees or migrants. These ethnic patterns are consistent with what is known about the epidemiology of HIV infection in pregnancy in the USA and in Europe [13,16,23].

Substantially more women from the European sites had been diagnosed with HIV prior to their current pregnancy than in the USA. Today, most identified HIV-infected women of child-bearing age are most likely diagnosed either as a result of antenatal testing or through more targeted testing of specific risk groups, such as injecting drug users or attenders of STD clinics. Parity was somewhat higher among the US women. Although there was a significantly higher prevalence of active IDU in Europe, this was only 3% in this setting. On the basis of these findings, the lower ascertainment of infection status among the US women before entry may reflect poorer access to or uptake of antenatal HIV testing in prior pregnancies or of HIV testing outside antenatal care, or alternatively, the possibility that the women from the USA acquired their HIV infection more recently (i.e. since a previous pregnancy), which is consistent with their younger age compared with the women from European sites, and their somewhat higher CD4 count at entry.

In terms of reproductive health, the HIV-infected women in the USA had a significantly higher prevalence of previous miscarriages than in Europe, double that in Europe, which may reflect a variety of factors including possible geographic/cultural differences with regard to reporting prior miscarriage as this was a self-reported variable. Although the women recruited in the US were younger than those enrolled in Europe, they had a higher parity than their European counterparts, whilst women from Europe had a significantly higher pregnancy termination rate; these findings may not only reflect cultural differences, but also access to reproductive care services [24].

The PACTG 316 trial spanned an important and dynamic era with regard to use of ART in pregnancy, both for maternal health and for preventing MTCT. Our findings reflect regional differences in use of combination ART, with more rapid uptake in the USA than in Europe [19,25,26]. In the Women and Infants Transmission Study, based in the USA and Puerto Rico, half of the pregnant women enrolled by 1998 were receiving HAART, increasing to more than 60% in 1999–2000 [27]. In Europe, although there was a steadily increasing uptake of combination therapy in pregnancy, in the absence of a randomised trial to show the efficacy of HAART in prevention of MTCT, some clinicians showed caution regarding prescribing HAART for women without symptomatic HIV disease, preferring to continue using the combination of zidovudine monotherapy according to the 076 regimen, elective caesarean delivery and avoidance of breastfeeding [28-31].

These parallel situations are apparent in the geographic differences seen here, with significantly more HAART use in the USA versus more monotherapy use in Europe. However, these differences are largely historical, and HAART now predominates in the treatment of pregnant women in Europe [32,33]. A fifth of women in Europe received the dual combination of zidovudine and lamivudine in pregnancy (data not shown); this partly reflects ongoing research in France at the time [34]. Furthermore, the lower prevalence of HAART use in European women may also reflect the non-eligibility of those women on HAART due to prior or current use of NVP-containing HAART, which was a popular regimen in Europe at the time [28]. Although the enrolled women from the US and Europe had similar CD4 counts and viral loads at entry, the US women had double the prevalence of symptomatic disease at entry (although this was low overall in both areas) and were less likely to have been diagnosed with HIV infection before the current pregnancy or to already be on ART at the time they became pregnant. Overall, 60% of women started ART for the first time in pregnancy and antenatal ART initiation was significantly earlier in the US than in Europe (18 weeks versus 27 weeks), probably reflecting timing of HIV diagnosis and/or management differences. By the time of delivery, although the two geographic groups remained immunologically similar, women from the USA were significantly less likely to have undetectable viral loads at delivery compared with women delivering in Europe, despite more women from the US receiving earlier and/or more potent ART. However, MTCT rates were similar between continents. In a previous analysis focused on women in the US sites of the PACTG 316 trial, black women (accounting for nearly two-thirds of the women enrolled) were significantly less likely to achieve undetectable viral loads by delivery than white women [35].

There was a considerably lower elective caesarean rate among the women delivering in the USA than in Europe, at 22% versus 61%. None of the explanatory variables (ethnicity, ART, maternal CD4 count or viral load) predicted likelihood of elective caesarean delivery among women from the USA. This is consistent with an individualised approach to elective caesarean delivery in this setting among HIV-infected women, with those with obstetric indications for elective caesarean most likely to constitute the majority of the elective caesarean group [36,37].

In Europe, guidelines current during the trial recommended universal offer of elective caesarean delivery to prevent MTCT [38-40]. However, as indicated by the sub-analysis of European women, receipt of HAART was associated with a significantly lower likelihood of having an elective caesarean delivery. This reflects the growing uncertainty from this time of the added benefit of elective caesarean delivery for women with undetectable plasma HIV RNA loads, and specifically concerns that the potential benefits in terms of reduced MTCT risk may be outweighed by the costs, such as the increased risk of post-partum complications and greater burden on health care services associated with elective caesarean delivery. This lack of consensus has continued in the absence of a definitive answer, with current policy and practice regarding mode of delivery varying considerably across Europe, although an increasing number of policies now recommend offering a vaginal delivery to women on successful HAART with undetectable viral load [41].

Concerns exist regarding the generalizability of results from HIV clinical trials, particularly those with stringent inclusion and exclusion criteria [42-44]. The PACTG 316 trial was designed with broad inclusion criteria, although the exclusion criteria of previous NNRTI-use may limit the generalizability of specific results here, as discussed above.

## Conclusion

These findings confirm that there are important historical differences between the HIV-infected pregnant populations in Western Europe and the USA, both in terms of the characteristics of the women and their obstetric and therapeutic management. Although the therapeutic management of pregnant HIV-infected women is now more uniform between the continents, with the predominance of HAART initiated from before or during pregnancy, significant population differences are likely to remain. This is of importance in interpreting results from previous research and in the design of future studies.

## Abbreviations

ART Antiretroviral therapy

HAART Highly active antiretroviral therapy

HIV Human immunodeficiency virus

IDU Injecting drug user

MTCT Mother-to-child transmission

PMTCT Prevention of mother-to-child transmission

RNA Ribonucleic acid

sdNVP Single dose nevirapine

## Competing interests

The author(s) declare that they have no competing interests.

## Authors' contributions

CKC, RDG, JS and MLN contributed to study concept and design. CKC, RDG, MLN, SF, LM, JS, RM, ID, DHW were involved in the acquisition of data. MLN, SF and CT drafted the manuscript. SH performed the statistical analyses. All authors critically revised the manuscript for important intellectual content. All authors read and approved the final manuscript.

## Pre-publication history

The pre-publication history for this paper can be accessed here:


